# Surface EMG analysis of weakness distribution in upper limb muscles post-stroke

**DOI:** 10.3389/fneur.2023.1135564

**Published:** 2023-04-25

**Authors:** Wenwen Lv, Kai Liu, Ping Zhou, Fei Huang, Zhiyuan Lu

**Affiliations:** ^1^School of Rehabilitation Medicine, Binzhou Medical University, Yantai, China; ^2^Department of Rehabilitation Medicine, University of Health and Rehabilitation Sciences Qingdao Hospital (Qingdao Municipal Hospital), Qingdao, China; ^3^School of Rehabilitation Science and Engineering, University of Health and Rehabilitation Sciences, Qingdao, China

**Keywords:** muscle weakness, weakness distribution, surface EMG, stroke, function assessment

## Abstract

Weakness is a common symptom after a stroke. This study aims to depict weakness distribution among forearm muscles given that joints in the upper limb are usually driven by a group of muscles. Multi-channel electromyography (EMG) was applied to assess the muscle group, and an EMG-based index was proposed to quantify the weakness of individual muscles. By using this method, four weakness distribution patterns were observed in extensor muscles from five out of eight subjects after stroke. Complex weakness distribution patterns were observed in flexor muscles from seven out of the eight subjects when they performed grasp, tripod pinch, and hook grip. The findings can help determine the weak muscles in a clinic and facilitate the development of appropriate interventions in stroke rehabilitation targeting specific muscle weakness.

## 1. Introduction

Stroke is a major cause of adult disability. Following a stroke, patients may suffer from a variety of physical symptoms, including spasticity, weakness, and impaired coordination. Weakness is one of the most common symptoms after stroke (1), which plays an important role in motor function deficiency and has a detrimental effect on the quality of life (2, 3). Assessment of muscle strength is, therefore, a fundamental component of stroke evaluation and treatment.

Evaluation of muscle strength after stroke can be done in various ways. Manual muscle testing (MMT) (4) is one of the well-known methods to assess weakness and is widely used in clinical settings because of its convenience. It is able to assess weakness distribution roughly such as the distribution between proximal and distal muscles (5). However, MMT is a subjective evaluation. Weakness can also be more objectively assessed by dynamometry, such as isokinetic dynamometry and handheld dynamometry (6), which can measure the output force or torque. Previous studies show that the output force is a representative measure of weakness, consistent with other assessments (7). However, limb movements (e.g., hand and finger movements) are usually generated by coordinating a group of muscles, so that it is difficult to assess each muscle's weakness and its distribution among different muscles by using these techniques.

Electromyography (EMG) signals recorded on the surface of the skin reflect muscle activities and contain rich information about physiological changes after stroke (8). EMG signals of affected muscles of stroke patients can be recorded even if they may not be able to implement or complete their intended movements (9, 10). By monitoring the activities of each individual muscle in a muscle group simultaneously using multi-channel EMG recordings, motion intents can be recognized based on the muscle activation patterns of stroke patients (10, 11). This has led to the success of EMG-driven exoskeletons for stroke rehabilitation (11–13). Surface EMG can also be used to predict muscle force given the strong relations between EMG and force (14–17). EMG amplitude of paretic muscles of stroke patients, measured as peak amplitude (18), average rectified value (ARV) (19), and root mean square (RMS) value (20), may alter compared with contralateral muscles.

Taking advantage of the usefulness and convenience of surface EMG, this study aims to explore the weakness distribution of upper limb paretic muscles after stroke. This was performed by assessing individual paretic muscle weakness using multi-channel surface EMG recording and analysis.

## 2. Methods

### 2.1. Subjects

Patients with chronic stroke and upper limb motion deficits were recruited from Qingdao Municipal Hospital and the local community. The screening was performed by an experienced physical therapist in order to exclude patients with the Modified Ashworth Scale (MAS) above 1+ or those with unmeasurable grip force. A total of eight stroke subjects (aged from 50 to 76 years, six male subjects and two female subjects, as shown in Table 1) participated in this study. Fugl-Meyer assessment was used to evaluate their hand and wrist function. Grip force was measured using a dynamometer (EH101, Camry, Guangdong, China).

**Table 1:** Subject demographics.

**Subjects**	**Gender**	**P**	**Age**	**MMT**		**FM**		**Grip force (kg)**	
				**Grasp**	**Open**	**Wrist**	**Hand**	**L**	**R**
S1	M	R	60	4	3	2	11	25.4	13.4
S2	M	L	72	4	4	10	13	31.8	36.7
S3	M	L	76	3	4	6	12	19.1	23.6
S4	F	L	65	3	3	6	10	16.5	21.7
S5	M	R	51	4	4	5	12	33.9	15.6
S6	M	R	70	4	4	7	10	30.6	13.8
S7	F	L	54	3	4	4	11	11.7	15.6
S8	M	L	50	4	3	7	11	23.2	37.1

P, paretic side; MMT, manual muscle test; FM, the Fugl-Meyer assessment scale of the wrist (total score: 10) and hand (total score: 14) of the paretic side.

### 2.2. Experimental protocols

The subject's two hands were tested individually in random order. A total of six EMG sensors (Trigno Avanti Sensor, Delsys, Natick, MA) were placed on the flexor digitorum superficialis (FDS), flexor digitorum profundus (FDP), medial head of extensor digitorum (EDM), lateral head of extensor digitorum (EDL), extensor pollicis longus (EPL), and extensor carpi radialis longus (ECR) muscle on the tested side. EMG signals were acquired using the Trigno system (Delsys, Natick, MA) at a sampling rate of 2 KHz. EMG sensors were first placed over the target muscle belly and subsequently adjusted in order to determine an optimal position where a relatively large EMG amplitude can be recorded. The position was confirmed by monitoring recorded EMG signals when the subject performed movements involving the target muscles. The skin was cleaned using alcohol pads prior to electrode placement.

During the experiment, the subject was seated comfortably on a chair with his/her arm rested on a table, elbow extension at 110°-120°, and the wrist placed in the neutral position. The subject performed each of the four motion patterns [grasp (squeezing a baseball), hand opening, tripod pinch (pressing a pinch gauge), and hook grip (squeezing a dynamometer)] 20 times (in 10 pairs) using the tested hand. In each pair, one movement was performed at maximum voluntary contraction (MVC) and the other one at medium (or comfortable) force, which was expected to be ~50% of MVC. This ratio was estimated based on the root mean square (RMS) value of EMG signals recorded from all the channels and was monitored by the experimenter, who gave verbal commands to the subject when the ratio fell outside of the recommended range. When performing the MVC task, the subject was asked to try his/her best and was given verbal encouragement (21). Each movement lasted for more than 5 s with isometric contraction for 3–5 s. The subject was given sufficient resting time (typically 5–10 s) after each movement in order to avoid muscle or mental fatigue.

### 2.3. Data preprocessing

Recorded EMG signals were filtered by a 20–450 Hz band-pass Butterworth filter and segmented by using RMS values calculated based on 100 ms moving windows. Specifically, a window was assigned to a movement if the RMS value across all the EMG channels within that window was greater than a given threshold (above three times the RMS value at rest or tuned by visual inspection), and vice versa. For each segment, in order to avoid transitive actions, the first 10 windows (i.e., 1,000 ms) and the last 10 windows were removed. RMS values of each EMG channel were then calculated for each pair of movements (denoted as $RMS_{m}^{mvc}$ for the movement performed at MVC, and $RMS_{m}^{medium}$ for the movement performed at medium force, and *m* refers to channel or muscle index). The weakness index for muscle *m*, denoted as *WI*_*m*_, was calculated as (1) and (2) for each pair. $RMS_{m}^{rest}$ refers to the RMS value of muscle *m* when the subject was completely relaxed.


$$\begin{array}{l} R_{m}=\frac{RMS_{m}^{mvc}-RMS_{m}^{rest}}{RMS_{m}^{medium}-RMS_{m}^{rest}}\;\left(1\right) \end{array}$$

$$\begin{array}{l} WI_{m}=\frac{R_{m}}{mean\left(R_{FDS},R_{FDP},R_{EDM},R_{EDL},R_{EPL},R_{ECR}\right)}\;\left(2\right) \end{array}$$

Values of *WI*_*FDS*_ and *WI*_*FDP*_ were calculated based on EMG signals recorded when performing grasp, tripod pinch, and hook grip. Values of *WI*_*EDM*_, *WI*_*EDL*_, and *WI*_*EPL*_ were calculated based on EMG signals recorded when performing hand opening. EMG signal processing including the calculation of WI was performed offline by using customized scripts in MATLAB (Math Works, Natick, MA).

### 2.4. Statistical analysis

Statistical analysis was performed using SPSS (version 23.0). The Wilcoxon rank sum test was employed to analyze WI values of the EDM, EDL, and EPL muscles between the paretic side and the contralateral side for each subject. Two-way ANOVA was applied to compare WI values of the FDS and FDP muscle obtained from each subject when performing three hand closing patterns (i.e., grasp, tripod pinch, and hook grip) by taking motion pattern and side (i.e., the paretic side and the contralateral side) as the two factors. Weakness was detected in muscle *m* if *WI*_*m*_ of the subject's paretic side was significantly smaller than that of his/her contralateral side. The significance level was set to 0.05.

## 3. Results

**Figure 1:**
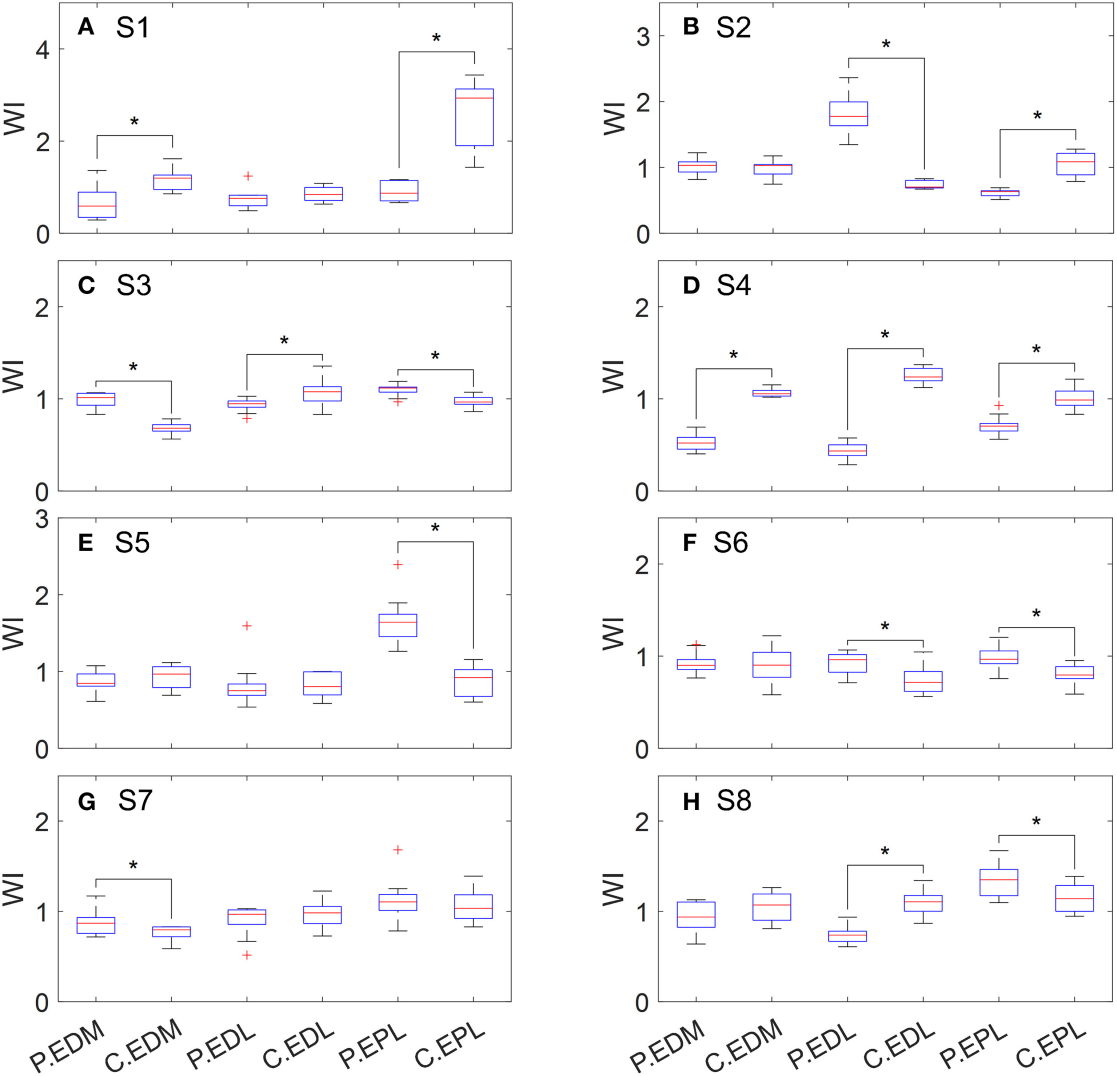
Distribution of WI values of extensor muscles when performing hand opening. P, paretic side; C, contralateral side. **(A)** S1, **(B)** S2, **(C)** S3, **(D)** S4, **(E)** S5, **(F)** S6, **(G)** S7, and **(H)** S8.

According to the values of *WI*_*EDM*_, *WI*_*EDL*_, and *WI*_*EPL*_, weakness was detected in these three extensor muscles in five out of the eight tested subjects (Table 2). Three subjects had weakness in a single muscle (either EDL or EPL), one subject had weakness in both EDM and EPL, and one subject had weakness in EDM, EDL, and EPL. The distribution of WI values is shown in Figure 1.

**Table 2:** Weakness distribution in forearm muscles.

**Subject**	**FDS**	**FDP**	**EDM**	**EDL**	**EPL**
S1			*p* = 0.024		*p* < 0.001
S2	*p* < 0.001				*p* < 0.001
S3	^*^	^*^		*p* = 0.013	
S4	*p* < 0.001	^*^	*p* < 0.001	*p* < 0.001	*p* < 0.001
S5	*p* < 0.001	*p* < 0.001			
S6		^*^			
S7	^*^				
S8	*p* < 0.001	^*^		*p* < 0.001	

* Weakness was observed when performing one or two grip patterns (using the Wilcoxon rank sum test) but not across the three grip patterns (using two-way ANOVA).

**Figure 2:**
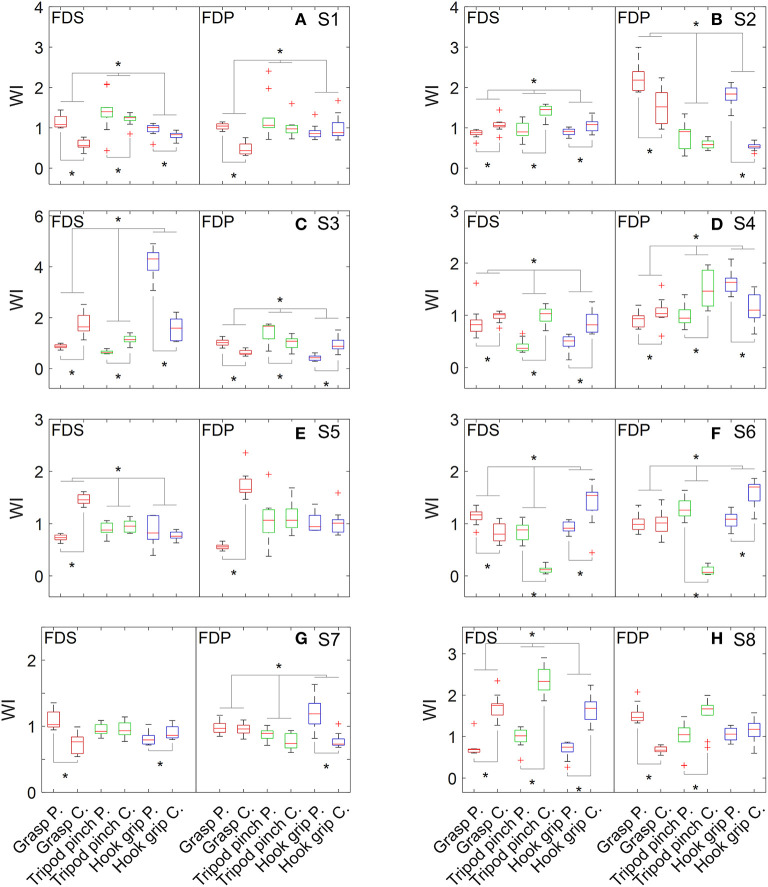
Distribution of WI values of flexor muscles when performing grasp, tripod pinch, and hook grip. **(A)** S1, **(B)** S2, **(C)** S3, **(D)** S4, **(E)** S5, **(F)** S6, **(G)** S7, and **(H)** S8.

As shown in Figure 2, *WI*_*FDS*_ obtained from different grip patterns was statistically different for seven subjects, and *WI*_*FDP*_ was statistically different across grip patterns for six subjects. Weakness was detected in FDS on six subjects, including two subjects whose weakness was observed only when performing one or two grip patterns (Table 2). Similarly, weakness was detected in FDP on five subjects, including four subjects whose weakness was observed only when performing one or two grip patterns. Specifically, four subjects had weakness in both the FDS and FDP muscle, two subjects had weakness only in the FDS muscle, and one subject had weakness only in the FDP muscle.

## 4. Discussion

According to the positive linear or curvilinear relation between force and EMG amplitude (which can be quantified using peak amplitude, ARV, RMS, etc.) observed in previous studies (18, 19, 22), force can be approximately estimated by surface EMG signals. Previous studies focusing on changes in the EMG-force relation usually targeted the first dorsal interosseous muscle (18, 19) and the bicep brachii muscle (22). These two muscles are considered to drive a single degree of freedom so that their force can be estimated by recording the output force or torque of the corresponding joint using a load cell. However, dexterous movements (e.g., grasp and pinch) are usually driven by the temporal coordination of a large group of muscles, and individuals with stroke may develop muscle compensation. Therefore, it is very difficult to obtain the precise force of each muscle, even when the output force of the limb (e.g., grip force) can be measured. This study applied a different strategy to detect weakness by evaluating each muscle's contribution to the explicit force of the limb, which was quantified as *WI*_*m*_. A significantly smaller contribution compared with the contralateral side was considered evidence of muscle weakness.

*WI*_*m*_ for each individual muscle is defined as the ratio of its *R*_*m*_ to the mean *R*_*m*_ value of all the muscles involved in the muscle group; thus, it is insensitive to either the absolute value of output force or the medium to MVC ratio even though *R*_*m*_ is dominated by the latter. The ECR muscle was included in the muscle group because it can enhance the grasp and grip forces by stabilizing the wrist and restraining the length of the flexor muscles.

Individual differences in weakness distribution were observed in both extensor and flexor muscles, reflecting the complex neuromuscular changes after stroke. Extensor muscles were assessed by performing one movement pattern (i.e., hand opening), while flexor muscles were assessed by performing three grip patterns that are frequently used in daily life. However, inconsistent weakness detection result was observed across different grip patterns on seven out of 16 flexor muscles. For example, the FDS of S3 appeared weak when performing grasp and tripod pinch but showed an opposite pattern when performing hook grip. It may be caused by abnormal movement patterns or impaired muscle coordination. It is also noted that *WI*_*m*_ of the paretic side could be greater than that of the contralateral side, which is likely attributed to a compensation mechanism for other weak muscles not examined in this study.

According to clinical assessments (MMT and Fugl-Meyer assessment, as shown in Table 1), all the subjects had weakness on the affected side. At least one weak muscle was detected in each subject (Table 2), which agreed with the assessments. It is worth noting that subject S2 had very good hand functions but experienced difficulties in performing thumb extension. It is usually caused by weakness of the EPL muscle, which was also detected by our method. Moreover, our results showed that he had weakness in the FDS muscle, and his relatively large grip force on the affected side was likely the result of FDP compensation. It is noted that the tested stroke subjects in this study were relatively high-functioning. More subjects need to be recruited in the future to improve the representativeness of stroke subjects.

Post-stroke weakness may be the result of several different factors including reduced cortico-spinal drive, intrinsic motor neuron property changes, trans-synaptic motor neuron degeneration, muscle contractile property changes, and muscle fiber disuse atrophy. In this study, conventional surface EMG electrode and amplitude feature were applied to assess muscle weakness as the result of complex neuropathic and myopathic changes post-stroke. Although conventional surface EMG can assess weakness distribution in a group of muscles, it is difficult to discriminate or quantify complex factors that contribute to muscle weakness. This remains a limitation of the current study. To address this limitation, more dedicated EMG techniques such as high-density surface EMG and intramuscular EMG are needed to evaluate different factors contributing to muscle weakness after stroke (23).

In summary, this study developed a convenient method for weakness assessment of individual muscles in a muscle group after a stroke. The method relies on conventional surface EMG recording and data analysis. Different weakness distribution patterns of upper limb muscles were observed after stroke. Therefore, we will recruit more subjects in the future to disclose the relation between these patterns and motor functions. The findings can help better understand stroke-induced weakness and function impairment and facilitate the development of appropriate interventions in stroke rehabilitation targeting specific muscle weakness.

## Data availability statement

The raw data supporting the conclusions of this article will be made available by the authors, without undue reservation.

## Ethics statement

The studies involving human participants were reviewed and approved by Ethics Committee of the University of Health and Rehabilitation Sciences. The patients/participants provided their written informed consent to participate in this study.

## Author contributions

ZL, PZ, and FH: study design and supervision. WL, KL, and ZL: data collection. WL, KL, PZ, FH, and ZL: data analysis and interpretation. WL and ZL: writing—original draft preparation. PZ and FH: writing—reviewing and editing. All authors have read and agreed to the published version of the manuscript.
